# Transcription Factor KLF14 and Metabolic Syndrome

**DOI:** 10.3389/fcvm.2020.00091

**Published:** 2020-05-27

**Authors:** Qianyi Yang, Mete Civelek

**Affiliations:** ^1^Center for Public Health Genomics, University of Virginia, Charlottesville, VA, United States; ^2^Department of Biomedical Engineering, University of Virginia, Charlottesville, VA, United States

**Keywords:** transcription factor, cardiometabolic diseases, human genetics, mouse models, transcriptional targets, sexual dimorphism

## Abstract

Metabolic syndrome (MetSyn) is a combination of metabolic abnormalities that lead to the development of cardiovascular disease (CVD) and Type 2 Diabetes (T2D). Although various criteria for defining MetSyn exist, common abnormalities include abdominal obesity, elevated serum triglyceride, insulin resistance, and blood glucose, decreased high-density lipoprotein cholesterol (HDL-C), and hypertension. MetSyn prevalence has been increasing with the rise of obesity worldwide, with significantly higher prevalence in women compared with men and in Hispanics compared with Whites. Affected individuals are at a higher risk of developing T2D (5-fold) and CVD (2-fold). Heritability estimates for individual components of MetSyn vary between 40 and 70%, suggesting a strong contribution of an individual's genetic makeup to disease pathology. The advent of next-generation sequencing technologies has enabled large-scale genome-wide association studies (GWAS) into the genetics underlying MetSyn pathogenesis. Several such studies have implicated the transcription factor KLF14, a member of the Krüpple-like factor family (KLF), in the development of metabolic diseases, including obesity, insulin resistance, and T2D. How KLF14 regulates these metabolic traits and increases the risk of developing T2D, atherosclerosis, and liver dysfunction is still unknown. There have been some debate and controversial results with regards to its expression profile and functionality in various tissues, and a systematic review of current knowledge on KLF14 is lacking. Here, we summarize the research progress made in understanding the function of KLF14 and describe common attributes of its biochemical, physiological, and pathophysiological roles. We also discuss the current challenges in understanding the role of KLF14 in metabolism and provide suggestions for future directions.

## Introduction

GWAS identified several genetic variants near the *KLF14* gene on chromosome 7 to be associated with a multitude of metabolic pathologies, including insulin resistance, T2D (1–3), and coronary artery disease (CAD) (4). The associations were stronger in females than in males (Table 1). While the initial GWAS was performed in people of European ancestry, many of the results have now been replicated in different ancestries (8–11). Expression quantitative trait locus studies linked the GWAS-associated genetic variants to the abundance of KLF14 transcript in adipose tissue (1, 12, 13). *KLF14* is a single exon imprinted gene, and only the allele inherited from the mother is expressed. As a transcription factor, the targets of KLF14 are largely unknown. In addition to genetic variants, studies showed environmental factors, such as diet, affected KLF14 expression in various metabolic tissues. In this review, we outline the current state of knowledge about KLF14 biology, and we provide suggestions for future studies to delineate its role in metabolism.

**Table 1:** Sex-specific and pleiotropic effects of the genetic variants at the KLF14 locus with cardiometabolic phenotypes.

		**Type 2 Diabetes (5)**	**BMI (6)**	**WHR (6)**	**WHRadjBMI (6)**	**Waist (7)**	**Hip (7)**	**HDL (2)**	**TG (2)**	**LDL (2)**
Combined analysis	Effect size	1.06 (1.05–1.08)	0.0083	0.0062	0.0125	−0.009	−0.017	−0.015	0.016	0.009
	*P* value	9.9 × 10^−18^	2.7 × 10^−7^	2.1 × 10^−4^	1.8 × 10^−13^	9.0 × 10^−3^	1.6 × 10^−6^	1.2 × 10^−15^	1.1 × 10^−6^	2.0 × 10^−2^
	N (case/control)	74,116/823,997	806,702	697,613	694,553	230,394	211,022	99,900	96,598	95,454
Female only	Effect size	1.09 (1.07–1.11)	0.0093	0.02	0.0282	0.008	−0.033	−0.042	0.036	0.018
	*P* value	8.9 × 10^−16^	1.8 × 10^−5^	9.7 × 10^−19^	3.6 × 10^−35^	8.0 × 10^−2^	9.9 × 10^−14^	3.5 × 10^−11^	1.0 × 10^−8^	2.0 × 10^−3^
	N (case/control)	30,049/434,331	434,716	381,115	379,449	126,971	117,288	62,816	59,473	61,803
Male Only	Effect size	1.04 (1.02–1.06)	0.0074	−0.0106	−0.0081	0.009	0.003	−0.034	0.013	0.01
	*P* value	1.0 × 10^−4^	1.6 × 10^−3^	2.2 × 10^−5^	1.3 × 10^−3^	4.0 × 10^−2^	0.5	9.9 × 10^−7^	4.0 × 10^−2^	0.19
	N (case/control)	41,842/383,763	74,693	16,682	315,238	03,616	93,919	7,745	35,288	36,840

*Effect sizes are with respect to the risk allele (C) of the index SNP, rs4731702. BMI, body mass index; WHR, waist-hip ratio; WHRadjBMI, BMI-adjusted WHR; HDL, high-density lipoprotein; TG, Triglycerides; LDL: low-density lipoprotein. The direction of effect size is presented with respect to the C (risk) allele of the index SNP in the locus (rs4731702). Association statistics for T2D, anthropometric traits, and lipids are from meta-analysis of GWAS from various populations. Summary statistics for rs4731702 across all studies were aggregated using fixed-effects meta-analysis with inverse-variance weighting of log-odds ratios or effect sizes. Effect sizes for T2D are odds ratios. Effect sizes for anthropometric traits and WHRadjBMI represent rank inverse normalization of the residuals after BMI was regressed out from WHR. Effect sizes for HDL, LDL, and fasting glucose are reported in mmol/l and for fasting glucose they are reported in pmol/L. Triglyceride (TG) results are log-transformed values*.

## Krüpple-Like Family of Transcription Factors

KLF14 is a member of the Krüpple-Like family of transcription factors which are responsible for modulation of gene expression in mammals (14, 15). There are 18 KLF proteins which are grouped into three distinct families [reviewed in (14)]. Group 1 KLFs primarily play a role in transcriptional repression while members of Group 2 function as activators of transcription. KLF14 belongs to Group 3 KLFs which typically play repressive roles by binding to DNA with Sin3A, another transcriptional co-repressor protein. However, KLF14 has been shown to activate transcription. KLF proteins contain conserved C2H2-type zinc finger domains in their C-termini which bind to GC-rich sites in the regulatory regions of target genes [reviewed in (14)]. In the absence of experiments that show direct binding of *KLFs* to the enhancers and promoters of target genes, it has been possible to predict common transcriptional targets using the structural similarities among KLFs (16). This strategy is especially important for KLF14 for which an antibody that can be used for immunoprecipitation for sequencing is not available.

The N-terminus regions of KLF proteins possess unique repetitive sequences that allow for binding with other protein partners, including co-activators, co-repressors, and histone-modifying enzymes (17). Sequence similarities in the amino-terminal domains lead to a correlation between structure and function. KLF14 has three C2H2-type zinc finger structural motifs at the C-terminus with the first and second domains comprising 25 amino acid residues and a shorter third domain containing 23 amino acid residues (18, 19). These three zinc finger domains can each recognize three DNA base pairs thereby contacting the gene regulatory region at three sites (20). There is evidence that the zinc finger domains have positively charged amino acids that may aid in nuclear localization. Several studies have examined the DNA binding site preference for KLFs and it appears that KLF proteins interact with similar GC-rich sequences or 5′-CACCC-3′ of the gene promoter regions (21, 22). KLF14 exerts its repressive activity on transcription by interacting with Sin3A, which acts as a transcriptional hub (23). Sin3A and Sin3B are large proteins that belong to a family of histone deacetylases that generally repress transcription by tethering to DNA. These large multi-domain Sin3 proteins integrate the function of transcriptional factors, such as KLF14, with chromatin-modifying activity.

The expression patterns of KLFs vary drastically in different tissues [reviewed in (14, 24)]; many members are widely expressed, including KLF6 (25), KLF7 (26), KLF9 (27), KLF10 (28), KLF11 (29), KLF13 (30) and KLF15 (31), whereas some exhibit tissue-specific expression. For example, KLF1 is mostly expressed in red blood cells and megakaryocytes (32, 33), KLF2 is largely present in white adipose tissue (34, 35), and KLF4 and KLF5 are predominant in the blood vessels, and white adipose tissue (36–39).

**Figure 1:**
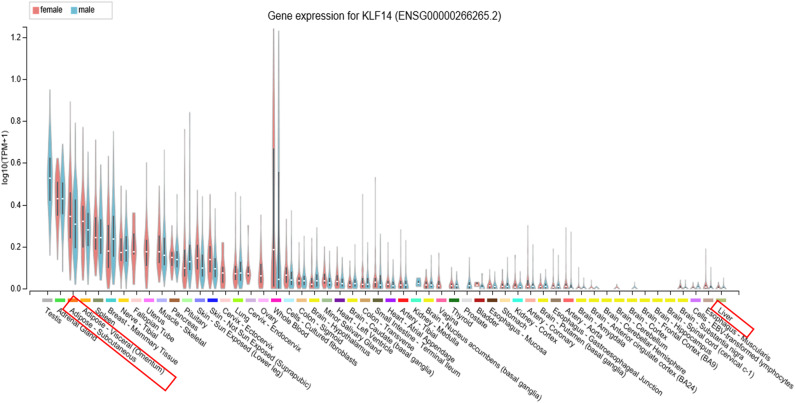
KLF14 expression in human tissues. Expression of KLF14 in 54 tissues or cell types from several hundred male and female donors was measured using RNA sequencing as part of the Genotype-Tissue Expression project. The results show widespread expression of KLF14 in various tissues, variation in the abundance of KLF14 in each human donor, and generally higher expression level in females compared to males. Image obtained from the GTEx Portal (40).

KLF14 is widely expressed in various tissues with higher expression in females compared to males (Figure 1). Given its wide-spread expression pattern, its role in various pathologies has been explored. Below we summarize the current state of knowledge of KLF14's role in various diseases.

## Human Genetic Evidence for the Role of KLF14 in Disease

The genetic variants associated with T2D and other metabolic phenotypes map to a region of 3–48 kb upstream of KLF14. GWAS-associated single nucleotide polymorphisms (SNPs) are also associated with the KLF14 expression level in adipose tissue (1, 12, 13). In other words, the GWAS signal for metabolic disorders and the expression quantitative trait locus for KLF14 are co-localized. Further, the same genetic variants are also associated with nearly 400 genes in *trans*. This is one of the largest *trans*-eQTL hotspots known in the human genome (41). GWAS of gene expression performed in the Multiple Tissue Human Expression Resource (MuTHER) and the Metabolic Syndrome in Men (METSIM) cohorts identified KLF14 as a master regulator of gene expression in subcutaneous adipose tissue (12, 41, 42). Interestingly, despite the nearly ubiquitous expression of KLF14 in many tissues and variation in its expression among humans (Figure 1) (43), the genetic variants regulate gene expression only in adipose tissue (1). This strongly suggests that disease-associated variants act in adipose tissue to increase the disease risk; however, given that adipose tissue contains a multitude of cell types, it is still not clear which cells are ultimately responsible for KLF14's effect on disease risk. *KLF14* is an imprinted gene and only the allele inherited from the mother is expressed (44); only the maternally inherited SNPs have significant associations with adipose tissue expression of *KLF14* (1). Small et al. have been able to fine-map the GWAS locus by using functional regulatory features from the Encyclopedia of DNA elements (ENCODE) (45) and the Roadmap Epigenomics Projects (46). They used chromatin-state maps generated by these projects to identify a ~1.6-kb enhancer region which is ~5 kb upstream of the *KLF14* transcription start site in adipose tissue (1). Overall, three distinct lines of evidence predict that the transcription factor *KLF14* is the causal gene in this GWAS locus and it functions in the adipose tissue: 1. association of *KLF14* expression in adipose tissue with T2D-associated SNPs but not in other tissues, 2. association of *KLF14* expression with maternal, but not paternal, alleles, and 3. the presence of an adipose-specific enhancer near the *KLF14* transcription start site in the locus associated with T2D.

In more than 70 thousand whole genome sequences from a diverse human population, only five potential loss-of-function (LoF) variants have been identified with very low frequency suggesting coding changes in KLF14 are under purifying selection (https://gnomad.broadinstitute.org/). However, to date, no LoF variants in *KLF14* have been shown to cause a rare disease, suggesting that regulatory variants that affect KLF14 expression are responsible for the GWAS findings.

## The Role of KLF14 in Cardiometabolic Disorders

T2D is the prevalent form of diabetes affecting ~10% of the US population. It is characterized by high blood sugar, and insulin resistance resulting in dysfunction of several organs. The expression pattern of most KLF members has been shown to be altered during disease progression with multiple lines of evidence suggesting that KLF14 is a key metabolic transcriptional regulator (42).

Insulin is a hormone produced in the pancreas that helps maintain the blood glucose homeostasis (47–49). Several members of the KLF family members, including KLF14, have confirmed roles in the regulation of insulin sensitivity [reviewed in (50)]. Yang et al. used high-fat diet (HFD)-fed mice and leptin receptor-deficient (db/db) mice that have impaired insulin sensitivity and showed that there is significant down-regulation of *Klf14* expression in skeletal muscle (51). At the molecular level, KLF14 activates PI3K/Akt signaling pathway to increase insulin sensitivity in the livers of HFD-fed mice and the *db/db* obese mice (51).

It has also been shown that KLF14 mediates lipid signaling (52). In cultured human endothelial cells, fibroblast growth factor 2 (FGF2) stimulation leads to the up-regulation of KLF14, which further induces sphingosine kinase 1 (SK1) expression (52). Mechanistically, KLF14 forms a complex with the coactivator p300 and then binds to a GC-rich region 633 bp upstream of the SK1 transcription start site (52) and activates the expression of SK1 (52). It has been shown that SK1 regulates adiposity by catalyzing the formation of lipid second messengers which leads to insulin resistance (53). Inhibition of SK1 resulted in accumulation of the lipid second messenger ceramides in liver cancer cells (54), while elevated muscle ceramides have been associated with muscle insulin resistance in obese humans (55). As such, some of the KLF14-mediated metabolic phenotypes may be attributed to regulation of lipid signaling via dysregulation of SK1 (50, 53). Below we summarize the studies that elucidated the role of KLF14 in metabolically active organs.

### The Role of KLF14 in Adipose Tissue

Excessive fat accumulation occurs when caloric intake is higher than energy expenditure. In response to the change in energy status, adipose tissue goes through rapid and dynamic remodeling by changing the number and/or size of adipocytes (56). Adipose tissues expand themselves to accommodate potential excess calories. In obesity, subcutaneous adipose tissue fails to expand appropriately in response to the excess energy intake, which leads to fat overflow and fat deposition in other ectopic sites, such as skeletal muscle, liver, and visceral fat depots. The consequence of this excess ectopic fat accumulation is insulin resistance (57–59). This shift in body fat distribution from subcutaneous to visceral is strongly associated with metabolic diseases (60–64). Adipocytes can be enlarged by excessive lipid deposition or generated from precursors through adipogenesis. Several members of KLFs play a role in energy homeostasis by regulating lipid and glucose metabolism, and many of them have been implicated in adipogenesis, including promoting [KLF4 (65, 66), KLF5 (37, 67, 68), KLF6 (69, 70), KLF8 (71), KLF9 (72), KLF13 (73), and KLF15 (74–77)] or inhibiting [KLF2 (78, 79), KLF3 (80), KLF7 (81, 82), KLF14 (1) and KLF16 (83)] adipocyte differentiation [reviewed in (84, 85)]. However, the role of KLF14 in hyperplasia is not known.

KLF14 appears to impact both pre-adipocyte differentiation to mature adipocytes and the function of mature adipocytes (1). To investigate the role of *KLF14* in adipogenesis, Small et al. isolated primary pre-adipocytes from human abdominal subcutaneous fat tissue and assayed *KLF14* levels over a 14-day differentiation time course. Cells from males and females had distinct patterns of expression throughout the differentiation (1). While *KLF14* expression did not change in male cells, it decreased and then increased as female adipocyte precursors matured into adipocytes. Interestingly, *KLF14* levels were higher in female subcutaneous adipocytes compared to male adipocytes throughout the time course (1). This was consistent with higher *KLF14* level in the adipose tissue of female donors compared to male donors both in the deCODE Icelandic cohort and the Genotype-Tissue Expression (GTEx) datasets (Figure 1) (1, 43). Lowering *KLF14* expression in cells from females prevented maturation as measured by the expression of adipogenesis markers and lipid accumulation suggesting a role of *KLF14* in adipocyte differentiation (1). H&E staining of subcutaneous adipose tissue biopsies showed that females who were homozygous for the T2D risk allele had adipocytes with larger cell surface area, suggesting that lower *KLF14* expression is associated with adipocyte dysfunction (1). These studies collectively suggested a role of *KLF14* in both adipogenesis and mature adipocyte size and function. However, a recent study that used machine learning to quantify adipocyte size from hundreds of histology images of adipose tissue did not find an association with adipocyte size at the *KLF14* locus (86).

In a recent study, Iwaya et al. analyzed the DNA methylation status of the *Klf14* promoter in adipose tissue to investigate if there was a correlation between aging and state of obesity, both of which are risk factors of T2D (87). Their study showed that, the expression of *Klf14* was reduced in adipose tissue of aged wild type mice and HFD-fed mice and consistent with this change in expression, *Klf14* promoter region was hypermethylated in adipose tissue in response to aging and HFD. Consistent with reduced *Klf14* expression, downstream target genes, many of which were associated with metabolic traits (88, 89), were also downregulated (87). In a study by Bacos et al. KLF14 hypermethylation was also associated with aging, insulin secretion and T2D (90). Taken together, these studies suggest that KLF14 loss-of-function via epigenetic regulation may trigger the onset of metabolic diseases. However, Argmann et al. showed that mice harboring whole-body germ-line deletion of *Klf14* were resilient to HFD-induced insulin resistance (91). Their results were inconsistent with the previously reported role of KLF14 in regulating HDL-C levels in the liver (92, 93). This is possibly due to the utilization of different mouse lines (Table 2). It is also likely that KLF14 function is redundant in mice and can be substituted with other KLFs. It is also possible that human and mouse KLF14 have different functions.

**Table 2:** Summary of published KLF14 mouse lines.

**Mouse line**	**Targeted region**	**Source**	**Diet**	**Sex studied**	**Phenotypes**	**Reference**
Whole-body *Klf14* knockout	1,035 bp between positions 30907660-30908694 of Chromosome 6 (Genome Build37), including the entire *Klf14* open reading frame	KOMP repository	Standard chow and 60% kcal high fat diet (switched at week 8)	Male	No difference in metabolic phenotypes; No difference was observed in whole-body *Klf14* KO mice plasma lipoprotein profile, cholesterol, HDL-C. No difference in insulin resistance and adipose gene expression change.	(91)
CRISPR-Cas9 whole-body knockout	A 7-bp frameshift insertion-deletion allele was introduced to the 5' end of the *Klf14* gene through CRISPR-Cas9	In house	45% kcal high fat diet (switched at week 18)	Male	Decrease in HDL-C level in *Klf14* whole-body knockout mice	(1)
KLF14 null mouse	The loxP sites were added ~1.6 kb upstream of the *Klf1*4 transcriptional start site and 300 bp downstream of the *Klf14* 3′-UTR separated by a total of 4.9 kb. Male offspring were crossed with *Sox2: CRE* transgenic females to generate a constitutive null allele	In house	Standard	Phenotypic analysis: female Gene expression analysis: male and female	Placenta is overgrown in *Klf14hetKO* mice who carry maternal *Klf14* allele (*Klf14matKO*). Fetal size remained unchanged. No morphological change in placenta layers. No abnormal lipid accumulation in *Klf14matKO* mice.	(94)
*Klf14*-KO mice	*Klf14* exon is disrupted by deleting 8-bp sequence using TALEN, which further disrupted the tenth amino acid and resulted in premature termination.	In house	Standard chow	NA	33.3% of *Klf14*-KO adult mice developed tumors in lung, spleen, and lymph nodes starting from 11-month-old. No spontaneous tumor was identified in other organs such as the heart, liver, kidney, breast, colon, and thymus. No obvious difference in body weight and serum lipids in KO mice.	(95)
Adipose-specific *Klf14* KO (Adn*Klf14*-KO)	Two LoxP sites were inserted at 3,415 bp upstream and 327 bp downstream of *Klf14* through CRISPR-Cas9. Offspring with two loxP sequences segregated on the same chromosome were crossed with *Adipoq-Cre* mice of the same genetic background	In house	Standard chow	Male and female	HDL-C was reduced in female Adn*Klf14*-KO mice while triglycerides were increased in males. Glucose tolerance and insulin sensitivity were impaired in both sexes	(1)
Liver-specific *Klf14* KO (*Klf14*-LKO)	*Klf14* exon flanked by LoxP sites (specific targeted region unknown), followed by germline transmission and *Flp* recombinase removal. Offspring were crossed with *Alb-Cre* transgenic mice (003574 from Jackson Laboratory)	In house	Standard chow	Male	Pooled blood serum was collected and analyzed by HPLC. Total cholesterol and triglyceride levels were comparable between WT and *Klf14-*LKO mice. HDL-C level was decreased in *Klf14-*LKO mice. ApoA-1 level was also decreased in hepatic *Klf14* KO mice liver.	(92)

### The Role of KLF14 in Liver Tissue

T2D-associated variants identified in GWAS also have strong associations with serum HDL-C and triglyceride levels (96, 97) (Table 1), specifically, T2D risk alleles at *KLF14* are associated with decreased HDL-C (1). However, the role of KLF14 in regulating cholesterol metabolism is not known. The liver is a specialized tissue comprising of mostly hepatocytes that regulate cholesterol and glucose metabolism. To maintain blood glucose levels, the liver provides glucose during low insulin and high glucagon conditions by both gluconeogenesis and glycogen breakdown (98). Insulin and glucagon are two hormones that work in concert to keep the blood glucose level balanced. When food is ingested insulin levels rise and glucagon levels dip; the liver removes excess glucose by breaking it down into pyruvate or using it for synthesizing glycogen (99).

It has been shown that hepatic KLF14 regulates glucose production in fasted and re-fed mice as well as diet-induced and genetically obese mice (93). Several lines of evidence suggest that increased KLF14 expression facilitates gluconeogenesis (93). Fasted animals had higher *Klf14* expression compared to *ad libitum* fed animals, and the expression levels returned to baseline levels after refeeding, suggesting a role of *Klf14* in gluconeogenesis (93). In the same study, Wang et al. also demonstrated that a high level of *Klf14* in wildtype C57BL/6J mice resulted in higher hepatic glucose production, high serum insulin levels and impaired glucose and insulin tolerance (93). shRNA-mediated silencing of *Klf14* in obese animals reduced blood glucose levels in fasting mice and increased glucose tolerance. *Klf14* overexpression in primary hepatocytes increased the transcript and protein levels of peroxisome proliferator-activated receptor-gamma coactivator 1-alpha (PGC-1α) a protein that stimulates glucose production. At the same time, *Klf14* knockdown reduced PGC-1α, thereby constraining glucose production (93). Taken together, these results suggested that KLF14 modulates hepatic glucose metabolism by regulating the activity of PGC-1α.

The liver regulates whole-body energy homeostasis in response to insulin stimulation (100). It has been suggested that higher *Klf14* expression protects the liver from insulin sensitivity. When Klf14 is overexpressed, there is increased glucose uptake concomitant with Akt kinase activation in mouse hepatoma cells (51). Overexpression of *Klf14* leads to increased transcription activation of PGC-1α, the histone deacetylase Sirtuin 1 and hepatocyte nuclear transcription factor 4α, all of which are insulin signaling-associated factors and play a role in glucose production in the liver (50, 51, 93, 101).

Guo et al. studied the role of KLF14 in regulating lipid metabolism in the liver and atherosclerosis using KLF14 overexpression and genetic inactivation in the livers of atherosclerosis mouse models (92). In contrast to Wang et al. they found that the KLF14 expression was reduced in the livers of HFD-fed and leptin-deficient (*ob/ob*) obese mice (92). Overexpression of KLF14 by injecting adenovirus encoding KLF14 via the tail vein increased HDL-C levels without affecting the levels of triglycerides, total cholesterol (TC), low-density lipoprotein cholesterol (LDL-C), and fasting blood glucose (92). Albumin Cre-mediated hepatic deletion of *Klf14* resulted in reduced HDL-C levels. Through promoter-bashing experiments and ChIP analysis, the authors showed that KLF14 directly binds to the promoter of apolipoprotein A-1 (ApoA1), which encodes the major protein component of HDL particles, and regulates its expression (92). Modulating KLF14 levels in hepatocytes *in vitro* or in mouse livers *in vivo* resulted in changes in the efflux capacity of HDL-C in agreement with KLF14's role in regulating ApoA1 expression (92). The authors also identified perhexiline from a drug screen, as a KLF14 activator, and used this drug to treat *ApoE-deficient* mice, which have very high total cholesterol levels and develop atherosclerosis. Administration of perhexiline increased hepatic KLF14 expression and HDL-C plasma levels which has a protective effect against atherosclerosis in *ApoE*-deficient mice (92). Lipids are transported in the blood serum as lipoproteins. HDL and LDL are the primary carriers of blood cholesterol. However, many laboratory animal models carry most cholesterol in HDL, while humans transport majority of cholesterol in LDL (102, 103). It is remarkable that there is consistency in the effect of the manipulation of KLF14 expression on HDL levels in humans and mice.

Wei et al. demonstrated that *ApoE*-deficient mice exhibited increased expression of KLF14 in their aortas compared to wild type controls when fed HFD or standard chow diet, which indicates a role for KLF14 in the vessel wall (104). Contrary to Guo et al. silencing KLF14 expression reduced circulating levels of inflammatory cytokines in HFD-fed *ApoE*-null mice (104). The authors noted decreased atherosclerotic lesions in the same *ApoE*-null mice likely brought upon by suppression of the M2 inflammatory phenotype of macrophages due to the inhibition of MAPK signaling, particularly extracellular signal-regulated kinase 1/2 and p38. These studies demonstrated a direct role of KLF14 in regulating atherosclerotic plaque and lesion formation independent of its risk factors (104).

It is not clear why there are discrepancies among the three studies which observed different effects of high-fat feeding and obesity on liver *Klf14* levels, fasting glucose, and the formation of atherosclerotic plaque. The conflicting expression profiles of *KLF14* reported in these studies can be attributed to the differences in the expression patterns of *KLF14* in different tissues. In addition, external factors, such as handling and stress, housing conditions, or methodological differences in measuring mRNA and protein levels may contribute to the observed discrepancy. In previous studies, we and others could not detect the expression of *Klf14* mRNA in human or mouse livers (1, 91, 105). The Genotype-Tissue Expression study, in which mRNA from 226 human livers were sequenced, also did not detect *KLF14* expression (Figure 1) (40). Our meta-analysis showed that risk allele is associated with decreased expression of adipose tissue KLF14 and decreased HDL-C levels and risk of developing T2D (Table 1), suggesting that higher levels of KLF14 may serve to protect against the development of adverse metabolic conditions. The association of genetic variants with KLF14 expression is only observed in adipose tissue suggesting that, instead of the liver, the metabolic effects of the risk variants are in the adipose tissue, which was not addressed in the aforementioned studies.

### The Role of KLF14 in Endothelial Cells

Endothelial cells (ECs), which line blood vessels, play significant roles in angiogenesis and the regulation of vascular function (106, 107). Hu et al. investigated the function of KLF14 in ECs. They showed that KLF14 is downregulated in mouse aortic ECs under acute and chronic inflammatory conditions (108). Overexpression of KLF14 suppressed inflammation in human ECs by inhibiting the pro-inflammatory response mediated by interleukin-1β (IL-1β) and tumor necrosis factor α (TNFα). *Klf14* knockout mice also exhibited increased expression of adhesion molecules in primary ECs when stimulated by IL-1 β (108). These results suggest that KLF14 protects ECs against inflammatory stresses. Mechanistically, KLF14 was shown to inhibit the expression of p65 subunit of the Nuclear Factor Associated Activator of Kappa B-Cells (NF-κB) signaling pathway, resulting in markedly reduced leukocyte adhesion to activated ECs (108). In addition, the KLF14 activator perhexiline was observed to induce KLF14 expression in ECs which in turn prevented leukocyte adhesion in mice (108). These results demonstrate that KLF14 inhibits the macrophage-mediated inflammatory response in ECs by transcriptionally inhibiting the NF-κB signaling pathway (108).

## The Role of KLF14 in Cancer and Placenta Development

The link between KLF proteins and regulation of cell cycle processes such as proliferation, differentiation, as well as cell adhesion and migration (15), and maintenance of pluripotency of stem cells (16) have been well established. More recently, several studies showed that KLFs also play important roles in cancer (14, 109) [reviewed in (15)]. Stacey *et al*. reported a new genomic locus associated with increased susceptibility to basal cell carcinoma (BCC) (110). This locus is 167 kb upstream of *KLF14* and is distinct from the T2D susceptibility locus (110). The authors showed that increased BCC incidence is coupled to the paternal allele, despite the observation that the maternal allele is responsible for *KLF14* expression (110). Fan et al. reported that disruption of *Klf14* gene in mice causes mitotic defects ranging from centrosome over-amplification, missegregation to chromosome aneuploidy, all of which contribute to the pathogenesis of tumors (95). Specifically, KLF14 negatively regulates Polo-like Kinase 4 (Plk4), a protein which when overexpressed leads to centrosome overamplification (111, 112). KLF14 downregulation is inversely correlated with Plk4 upregulation in many cancers; these findings were affirmed by data analysis from cancer microarray database Oncomine (www.oncomine.org) (95). The authors also demonstrated that it is essential to maintain KLF14 activity within a normal range for cells to keep their genomic integrity. Deletion of KLF14 using transcription activator-like effector nucleases (TALEN) caused genomic instability in mice, such as chromosomal aneuploidy and chromosome missegregation, whereas KLF14 overexpression induced a host of mitotic defects that proceed with initiation of apoptotic programs and end with cell death (95). Consistent with a negative regulatory role of KLF14 in relation to *Plk4*, overexpression of Plk4 lead to chromosomal instability in gastric cancer whereas Plk4-null mouse embryos had a remarkable increase of mitotic cells (95). It is known that mitotic catastrophe is induced as a cellular mechanism to avoid genome instability (113) and as such, it is plausible that cells undergoing stress enhance expression of KLF14 subsequently initiating mitotic catastrophe to circumvent genomic instability in dividing cells. They proposed that KLF14 plays a tumor suppressor role, since *Klf14* transcription is significantly downregulated in multiple types of cancers (95). As such, KLF14 presents as a new biomarker or potential target for cancer therapies.

Parker-Katiraee et al. identified *KLF14* as a maternally imprinted gene with near-ubiquitous expression in mice and human (105). The authors observed greater *KLF14* expression levels in embryonic tissues than in adult tissues, indicating a role in embryogenesis (105), which is consistent with the finding that several imprinted genes have been observed to regulate growth and embryonic development [reviewed in (114, 115)]. It has been proposed that maternally expressed genes are responsible for increasing the mothers' survival rate by suppressing fetal growth, whereas paternally expressed genes augment fetal growth and development to guarantee the viability of their genetic offspring (116). This would implicate KLF14 to play a repressive role in fetal and/or placental development and growth. In addition to adipose tissue, KLF14 is also highly expressed in the placenta (43). A recent study by Koppes et al. in mice showed that offsprings from *Klf14*-deficient mothers showed an overgrowth of the placenta in late gestation, however, fetal weight remained unchanged (94). RNA sequencing of placentas from *Klf14*-deficient and wild type mice identified 21 differentially expressed genes such as *Col26a1* (94). Consistent with KLF14's implicated role as a transcriptional repressor (1, 117), the loss of *Klf14* activated many placental genes. However, none of the significantly differentially expressed genes overlapped with previously reported adipose tissue *KLF14 trans*-regulated target genes nor with previously reported liver tissue targets in *Klf14*-deficient mice (1, 52, 92, 93), possibly due to distinct transcriptional regulation profiles in different tissues.

## Mouse Models of *KLF14*

Several mouse studies showed conflicting results, for example, for the role of liver *Klf14* in atherosclerosis. One major factor that may be causing the discrepancies in these studies is the utilization of distinct mouse lines and experimental designs, which are different in many aspects, including targeted regions, targeting approaches, tests performed, and phenotypes observed. We provide a summary of the different *Klf14* mouse lines which have been reported in the literature (Table 2). Thus far, there are four *Klf14* whole-body knockout mice generated by four different groups. Argmann et al. obtained *Klf14tm1.1(KOMP)Vlcg* mouse sperm from the Knockout Mouse Project (KOMP) repository (91). In these mice, 1035 bp between positions 30907660-30908694 on chromosome 6 (Genome Build 37), which includes the entire *Klf14* open reading frame, was deleted through homologous recombination (91). The authors reported no significant differences in metabolic phenotypes comparing these knockout animals to their wild type littermate controls. In contrast to previous reports, no changes in HDL-C, cholesterol and ApoA-I plasma levels were observed in *Klf14*-deficient mice. However, the authors did not study female and male mice separately, a consequential omission given that in humans, KLF14 plays sexual-dimorphic roles. The second whole-body knockout mouse was generated by the Karpe Lab by inserting a 7 bp frameshift sequence using CRISPR-Cas9 at the beginning of the *Klf14* gene to mitigate the disruption of neighboring regulatory regions (1). They reported that serum HDL-C level was reduced following the disruption of *Klf14* in these mice when fed with HFD. The third *Klf14*-null mouse line was reported recently by Koppes et al. (94). This mouse line was generated by flanking the *Klf14* gene with two loxP sites, separated by a total of 4.9 kb. This strategy targets a much bigger region than other whole-body knockout mouse lines. It was not clear if the neighboring gene expression was analyzed to ensure adjacent regulatory elements were not disrupted. This group reported no abnormal lipid accumulation in the placentas of *Klf14-*deficient mice. Lastly, Fan et al. generated a *Klf14*-KO mouse line by deleting an 8 bp sequence using TALEN, which resulted in the disruption of the tenth amino acid and premature termination (95). These *Klf14*-KO mice did not display abnormal changes in their body weight or serum lipids. Instead, they developed spontaneous tumors at various locations. However, spontaneous tumorigenesis was not reported in other *Klf14* whole-body knockout mice. Chen and Musunuru groups generated two tissue-specific *Klf14* knockout mouse lines: a liver-specific knockout (*Klf14*-LKO) (92) and an adipose-specific knockout (Adn*Klf14*-KO) (1). Both lines were generated by inserting two LoxP sites flanking the *Klf14* gene, followed by crossing with mice harboring liver (*Alb*)- or adipose (*Adipoq*)-specific Cre transgenes. Adn*Klf14*-KO mice showed decreased HDL-C in females and increased triglycerides in males with both sexes exhibiting impaired glucose tolerance and insulin sensitivity (1). *Klf14*-LKO mice showed decreased HDL-C levels but no change in TC and triglyceride levels in male mice (92). Female mice were not studied. While mouse models are useful to establish causality, they do not capture the subtle differences in KLF14 abundance among individuals as observed in human studies. Further, developmental compensation by related KLFs cannot be neglected as a possibility for the lack of an observed metabolic phenotype in some studies or conflicting results in others.

## Transcriptional Targets of KLF14

Direct transcriptional targets of KLF14 are largely unknown. Several studies identified individual transcriptional targets of KLF14 using multiple approaches. Sarmento et al. showed that KLF14 regulated regulatory T-cell differentiation via chromatin remodeling at the FOXP3 Treg-specific demethylation region based on ChIP analysis suggesting that FOXP3 is a potential target of KLF14 (118). Truty et al. showed that KLF14 exerts repressive pressure on the TGFβRII promoter by binding to GC-rich binding motif and by competing with Sp1 using ChIP assays, site-directed mutagenesis, and electromobility shift (119). They further observed that the N-terminus region of KLF14 binds with Sin3A to form a co-repressor complex that represses the *TGF*β*RII* promoter, which is consistent with the fact that KLF14 employs the chromatin-modifying capability of Sin3A to repress transcription (119). Guo et al. showed that ApoA-I is decreased in *Klf14* liver-specific knockout mouse model, indicating KLF14-mediated transcriptional regulation of ApoA-I (92). They demonstrated that the human *APOA1* promoter region contains two KLF14 binding sites (CACCC box). ChIP assay showed that KLF14 binds to regions containing the CACCC box, demonstrating that APOA1 is a functional KLF14 transcriptional target. De Assuncao et al. first demonstrated the transcriptional activation role of KLF14 by identifying Sphingosine kinase 1 (SK1) as its transcriptional target (52). *SK1* mRNA levels were observed to decrease following *KLF14* siRNA treatment in endothelial cells. Specifically, using ChIP and promoter binding assays, the group demonstrated that KLF14 binds to the promoter region of SK1 and represses its transcription (52). Wei et al. have shown that adenovirus-mediated *KLF14* knockdown is significantly associated with the increased level of MAPK proteins including ERK1/2 and p38, stress-induced signaling pathways that are associated with the transcription of some proinflammatory factors and pathogenesis of atherosclerosis (104). However, whether or not P38 MAPK or ERK1/2 are transcriptional targets of KLF14 still require further validation.

Recent advances in human genetic and bioinformatic approaches have been employed to identify genetics variants that are associated with *KLF14* in adipose tissue. The same genetic variants are also associated with nearly 400 genes in *trans*. The promoter regions of the *trans*-regulated genes were enriched for the *KLF14* binding motif (1), although, there is conflicting evidence about the exact binding motif of *KLF14* (120, 121). Many of these *trans*-genes have been demonstrated to regulate MetSyn. For example, SLC2A4 impairs glucose tolerance (122); STARD10 impairs glucose-stimulated insulin secretion (123, 124); and the IDE regulates the degradation of insulin (125). Many of the putative target genes were also linked to a range of metabolic characteristics, such as waist-to-hip ratio, body mass index (BMI), cholesterol, insulin, and blood glucose levels. These putative transcription targets are waiting for further experimental validation.

## Conclusions and Future Studies

**Figure 2:**
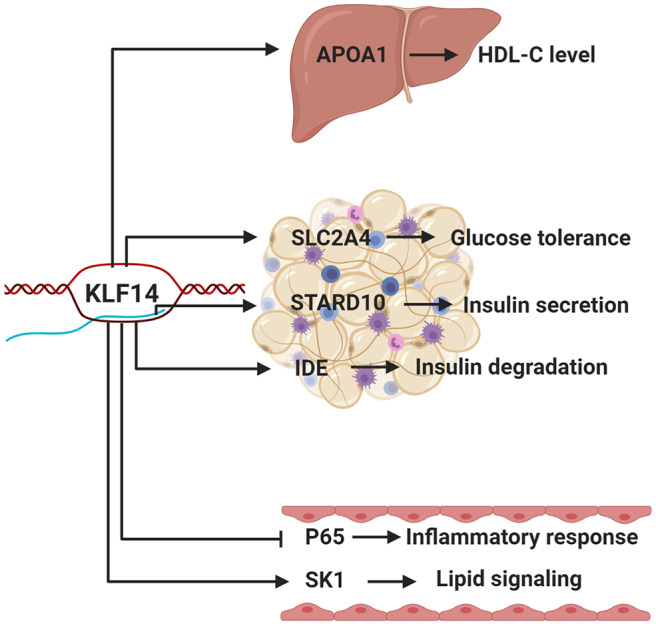
The function of KLF14 in metabolic tissues. Putative KLF14 transcription targets in different metabolically active tissues/organs, as well as their potential roles, are shown. Human genetics studies show that risk allele is associated with lower KLF14 expression in adipose tissue. Schematic depicts the *KLF14* risk allele.

It has been nearly 20 years since Klf14 was first described and cloned (19). In the intervening years, its role in metabolic disorders and cancer has been established through a series of human genetics and mouse studies (Figure 2). However, conflicting results in mouse models show the need for additional carefully performed studies to elucidate its role in human disease.

The genetic variants of the *KLF14* locus in the human genome are significantly associated with a multitude of MetSyn-related traits and disorders. However, the causal variant(s) that is responsible for metabolic disorders, increased T2D/CAD risk, and changes in KLF14 expression remains elusive. These variants appear to regulate KLF14 expression only in adipose tissue and the cause of this tissue specificity is not known. *Trans*-ethnic fine-mapping studies, massively parallel reporter assays, or creating cell lines with different alleles on an isogenic background may help to identify the causal variant(s) in the locus (126). However, it will be more difficult to identify the tissue-specific effect of the causal variants since adipose tissue consists of a multitude of cells.

It is also crucial to identify the direct KLF14 targets to understand how KLF14 functions. A systematic approach identifying the direct KLF14 targets is lacking. Approximately half of the nearly 400 genes identified in the Small et al. study (1) had KLF14 binding sites in their promoter/enhancer regions, but having a binding site is not enough evidence to show that the transcription factor actually binds to that sequence to regulate target gene expression. The currently preferred method for identifying targets of transcriptional factors is to reduce or induce their expression using shRNA or overexpression vectors, followed by the identification of differentially expressed genes. A subset of genes with a consensus binding sequence near the transcription start sites is then defined as transcriptional targets. However, in this approach, the effects of prolonged modulation of transcription factor expression are dominated by secondary (and beyond) effects and it is impossible to discern primary transcriptional targets. Furthermore, proximal transcription factor binding alone is not sufficient to modulate gene expression (117). To avoid these complications, novel approaches that allow the identification of primary KLF14 targets by determining the impact of rapid KLF14 depletion on nascent RNA transcription should be employed (127–129).

Finally, the sex-biased effects of KLF14 on metabolic phenotypes are intriguing, especially since these effects do not appear to be hormonally-driven (1). Multiple studies show that KLF14 expression is higher in females, but the effect of the genetic variants on KLF14 is similar in males and females (1, 12). There is also a great deal of overlap between the adipose tissue genes whose expression is associated with the KLF14 locus in *trans* in males and females (1, 12). However, this is based on a study that identified the overlap in *trans*-regulated genes in two distinct studies. A more careful approach would be to calculate the association of all the adipose tissue-expressed genes in males and females recruited from the same population and whose gene expression was measured with the same method. GTEx study provides an opportunity for such a study to be performed (43). It is also possible that KLF14 shows an effect based on a threshold of abundance and since females have more KLF14 expression, the metabolic effects are more pronounced.

In summary, based on human genetics findings, inducing KLF14 expression in adipose tissue would be predicted to be metabolically beneficial, at least in females. However, greater insights into the mechanistic pathways that engender KLF14-mediated effects on adipose tissue are warranted to comprehensively evaluate its potential as a therapeutic target. It has been shown that perhexiline, a small drug that is approved for clinical use in many countries except the United States, to treat angina and heart failure, can effectively induce KLF14 expression in the liver while reducing atherosclerosis in *ApoE-null* mice (92). However, chronic use of perhexiline is associated with hepatotoxicity and neurotoxicity, which is why it has been removed from the markets of several countries. To this end, the derivatives of perhexiline could be potential substitutes (130). Another alternative to avoid unwanted side effects would be to deliver the drug directly to adipose tissue. Xue et al. have developed nanoparticles that can deliver anti-obesity drugs directly to adipose tissue (131). This approach can be optimized to deliver KLF14 agonists to improve adipose tissue function in metabolic disorders.

## Author Contributions

QY and MC planned and wrote the manuscript.

## Conflict of Interest

The authors declare that the research was conducted in the absence of any commercial or financial relationships that could be construed as a potential conflict of interest.
